# Emergent Invasive Group A *Streptococcus dysgalactiae* subsp. *equisimilis*, United States, 2015–2018

**DOI:** 10.3201/eid2508.181758

**Published:** 2019-08

**Authors:** Sopio Chochua, Joy Rivers, Saundra Mathis, Zhongya Li, Srinivasan Velusamy, Lesley McGee, Chris Van Beneden, Yuan Li, Benjamin J. Metcalf, Bernard Beall

**Affiliations:** Centers for Disease Control and Prevention, Atlanta, Georgia, USA

**Keywords:** invasive group A Streptococcus, *Streptococcus dysgalactiae*, *Streptococcus pyogenes*, *Streptococccus equisimilis*, Lancefield group A antigen, multilocus sequence type, bacteria, bacterial infections, Active Bacterial Core surveillance, ABCs, GAS, streptococci

## Abstract

The term group A *Streptococcus* is considered synonymous for the species *Streptococcus pyogenes*. We describe an emergent invasive *S. dysgalactiae* subspecies *equisimilis* lineage that obtained the group A antigen through a single ancestral recombination event between a group C *S. dysgalactiae* subsp. *equisimilis* strain and a group A *S. pyogenes* strain.

The Centers for Disease Control and Prevention’s Active Bacterial Core surveillance (ABCs) performs population-based surveillance of invasive group A *Streptococcus* (GAS) infections. Isolates collected from a population of ≈34 million persons are subjected to whole-genome sequence (WGS)–based characterization. We recently detected group A carbohydrate-positive *S. dysgalactiae* subsp. *equisimilis* (SE) isolates employing the *gacI* (*1*) sequence query within our bioinformatics pipeline (*2*). GAS is considered synonymous with *S. pyogenes*, rare occurrences of group A SE have been noted (*3*,*4*).

## The Study

**Figure 1:**
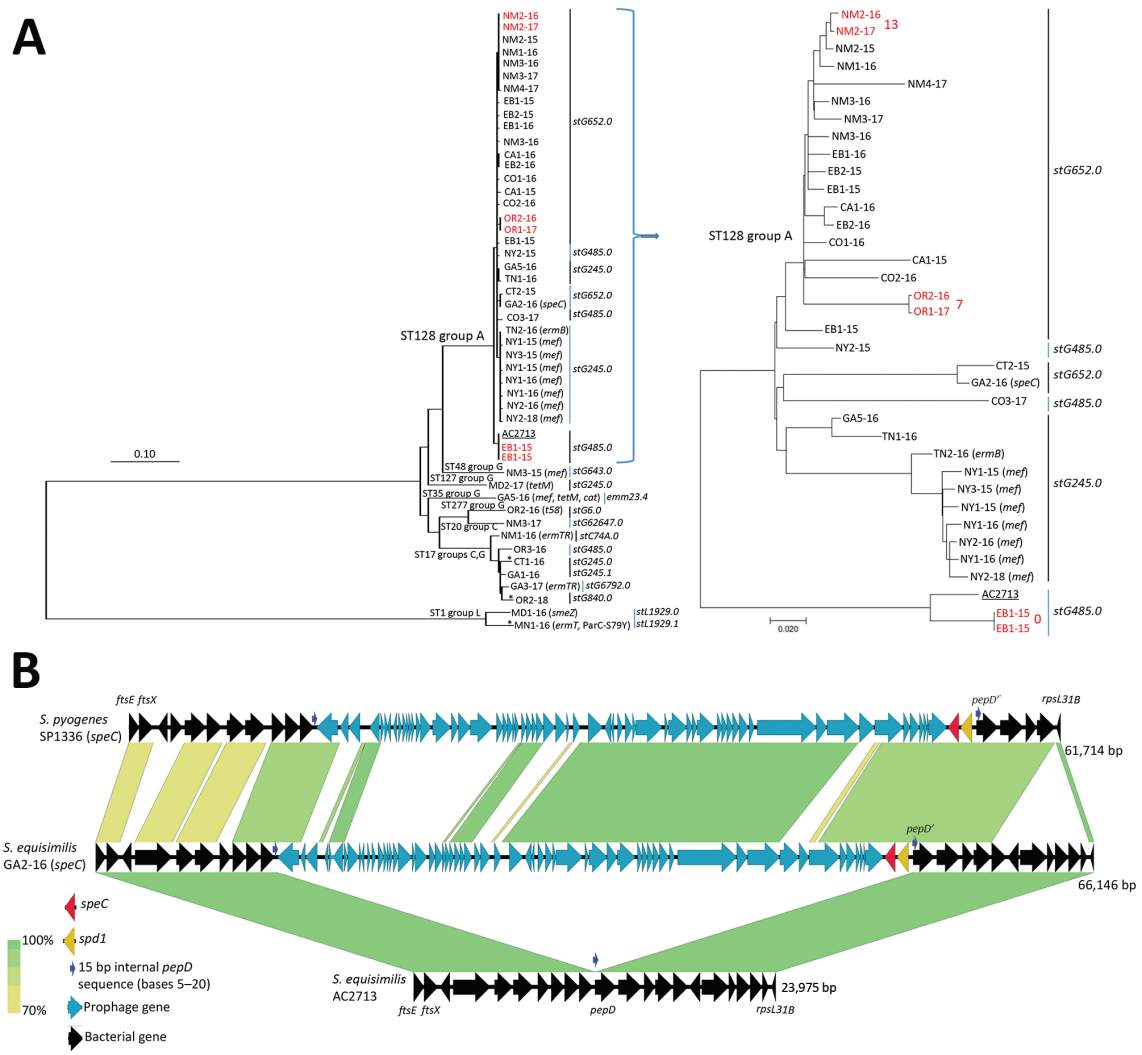
Analyses of invasive group A *Streptococcus dysgalactiae* subspecies *equisimilis* and conserved genomic *pepD* gene insertion site of highly related exotoxin *speC* gene–containing prophages found within group A ST128 *S. equisimilis* strain and *S. pyogenes* strain SP1336. Methods are described in the Appendix. A) Phylogenetic tree of 35 invasive group A *S. dysgalactiae* subsp. *equisimilis* (GAS/SE/MLST128 [ST128] complex) isolates and 13 unrelated group C, G, and L SE isolates recovered through the Centers for Disease Control and Prevention’s Active Bacterial Core surveillance during January 1, 2015–November 1, 2018. Trees are drawn to scale; branch lengths indicate number of substitutions per site. Surveillance areas (https://www.cdc.gov/abcs/reports-findings/surv-reports.html) are indicated: EB, East Bay San Francisco area, California; NY, New York; NM, New Mexico; CA, San Francisco Bay area, California; OR, Oregon; CO, Colorado; GA, Georgia; CT, Connecticut. Different counties and years of isolation are indicated (e.g., EB1–15 indicates county 1 in East Bay area and year 2015). The left tree depicts all 49 isolates and the right includes only the subset of the 36 GAS/ST128/SE (also including GAS/ST128/SE described by Brandt et al. [*3*] and assigned GenBank accession no. HE858529). Three pairs of isolates differing by 13 or fewer single-nucleotide polymorphisms are shown in red. Single-locus variants of the indicated multilocus sequence types are indicated with asterisks. B) Conserved genomic *pepD* gene insertion site of highly related exotoxin *speC* gene–containing prophages found within group A ST128 *S. equisimilis* strain (middle) and *S. pyogenes* strain SP1336 (GenBank accession no. CP031738). The nonfunctional *pepD* structural genes lacking bases 1–4 are depicted in the 2 prophage-containing strains. Nucleotide sequence identity is scaled from 70% (yellow) to 100% (green). The *S. equisimilis* prophage also contained the virulence-associated DNase gene *spd1* as shown and previously described for the depicted SP1336 phage shown (*8*). Within both species, the *pepD* insertion site lies within a region between the conserved bacterial cell division genes *ftsE/ftsX* and the small ribosomal protein gene *rpsL31b* (GenBank accession no. for *S. equisimilis* AC2713 is HE858529).

During January 1, 2015–November 1, 2018, a total of 5,480 ABCs GAS isolates were subjected to WGS. We identified 35 atypical *gacI*-positive isolates; each yielded 1 of the M protein gene (*emm*) subtypes *stG245.0*, *stG485.0*, or *stG652.0* commonly associated with SE (*4*–*6*). These 35 isolates lacked multilocus sequence types (MLSTs) inclusive of known *S. pyogenes* allelic designations. Lancefield grouping (*7*) and MLST (https://pubmlst.org/sdysgalactiae) (*6*) revealed the 35 isolates were serologically group A and MLST sequence type (ST) 128 (GAS/ST128/SE). We received 13 additional SE isolates recovered through ABCs GAS surveillance during this period that were found to be non–group A isolates (9 group G, 2 group C, and 2 group L) with MLSTs unrelated to ST128 (Figure 1). According to our normal protocol, these 13 non–group A SE isolates and 2 group G *S. canis* isolates that we also received were removed from the ABCs GAS database.

**Figure 2:**
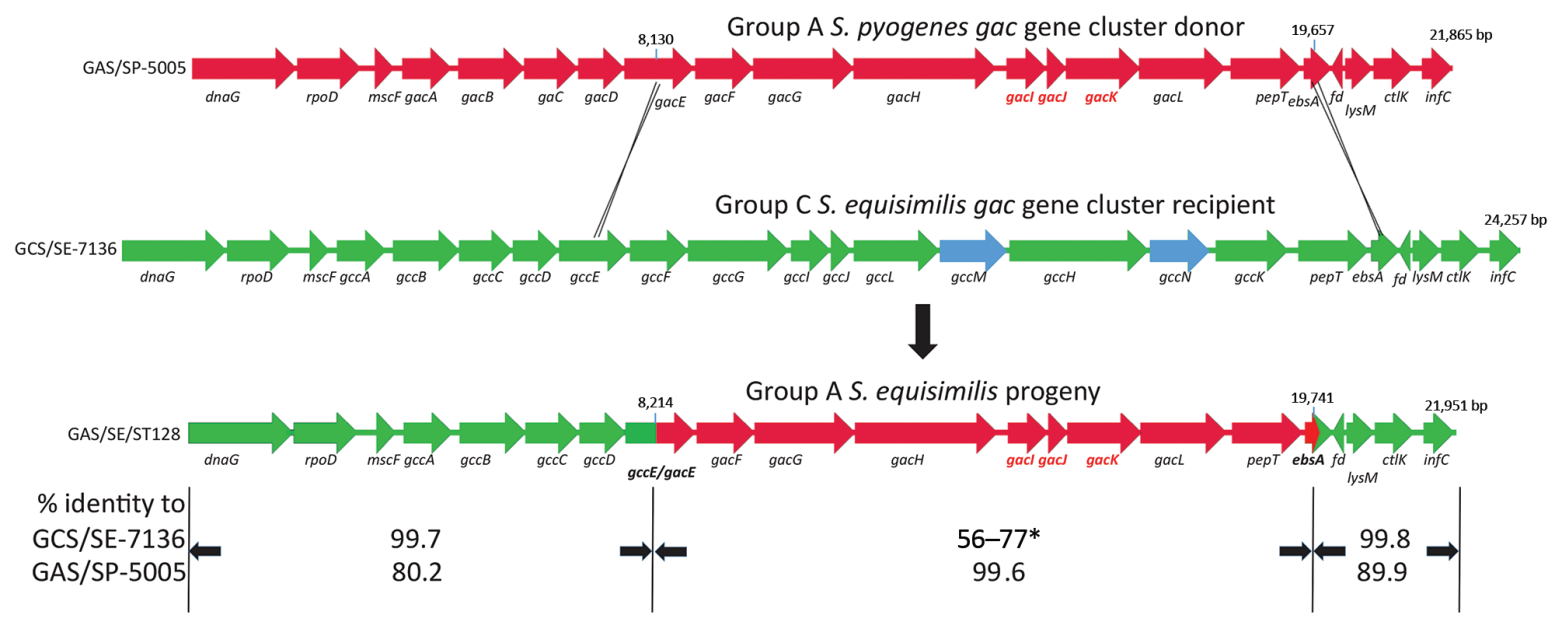
Ancestral recombination event depicting *Streptococcus pyogenes* group A carbohydrate gene donor (GAS/SP-5005; GenBank accession no. NC007297), group C *S. dysgalactiae* subsp. *equisimilis* recipient (GCS/SE7136; GenBank accession no. NCTC7136), and progeny group A *S. dysgalactiae* subsp. *equisimilis* progeny (GAS/SE/ST128) described in study of emergent invasive group A *Streptococcus dysgalactiae* subspecies *equisimilis*, United States, 2015–2018. The deduced crossover points between the group A gene cluster (red) donor and group C (green) recipient strains are shown. The 3 genes required for inclusion of the immunodominant *N-*acetylglucosamine side chain within the group A carbohydrate (*gacI, gacJ*, and *gacK*) are shown in red. The coordinates of the fragment transferred that is highly conserved between the donor and the progeny are indicated. The length of the 3 genomic regions are indicated. The *gacE/gccE* and *ebsA* genes are shown as green/red hybrids. The extra *gcc* cluster genes not conserved within the *gac* cluster are shown in blue. The relative sequence identities of the 3 different regions of progeny (bottom) *gac* cluster genes with the group A *S. pyogenes* donor (top) and group C *S. equisimilis* recipient (middle) are indicated. The middle segment (asterisk) indicates a range of 56%–77% sequence identity between each of the 8 structural genes (*gacF–pepT*) that were received intact from the *S. pyogenes* donor. The *gac* cluster genes are described in more detail in van Sorge et al. (*1*). Gene assignments are as follows: *dnaG*, DNA primase; *rpoD*, major RNA polymerase sigma factor; *mscF*, metal sulfur complex assembly factor; *gacA-L*, group A carbohydrate biosynthetic genes (putative functions described in van Sorge et al. [*1*]); *gccA-N*, group C carbohydrate biosynthetic genes. *gccA-L* are functional homologs of *gacA-L. gccM* and *gccN* putatively encode an additional glycosyl transferase and UDP-monosaccharide 4-epimerase, respectively; *ebsA*, pore-forming protein; *fd*, ferredoxin (complement strand); *ctlK*, cytidylate kinase; *infC*, translation initiation factor IF-3.

The Lancefield group A carbohydrate consists of a polyrhamnose chain with an immunodominant *N-*acetylglucosamine side chain (*9*) that functions in GAS pathogenesis (*1*). The group C carbohydrate also has a polyrhamnose backbone; however, its immunodominant side chain is the dissaccharide *N-*acetylglucosaminosyl-*N-*acetylglucosamine (*9*). Genomic comparison of the 12 gene group A carbohydrate synthetic cluster *gacA-L* (*1*) from *S. pyogenes* with the corresponding regions of the 35 GAS/ST128/SE revealed an upstream crossover point within the *S. pyogenes gacE* ABC transporter gene and a downstream crossover point within *ebsA* (Figure 2). The ancestral recipient SE strain was implicated as group C *S. equisimilis* (GCS/SE) by virtue of the near-identical sequence of the 1,363-bp sequence within GAS/ST128/SE encompassing sections of *gacD* and *gacE* homologs (designated as *gccD* and *gccE*) (Figure 2) with GCS and the marked divergence of this 1,363-bp sequence from group G SE (data not shown). This sequence is immediately adjacent to the upstream crossover point shown between *S. pyogenes* and GCS/SE (SP-5005 and SE-7136; Figure 2). We also found these same crossover points within the group carbohydrate gene cluster of the available genomic sequence from the previously described invasive GAS/SE strain AC-2713 recovered in 1999 (*3*). Subsequent genomic analysis revealed AC-2713 to be ST128 and *emm* type *stG485.0*. Phylogenetic analysis revealed that AC-2713 differed by 126 single-nucleotide polymorphisms from a pair of genetically indistinguishable GAS/ST128/SE recovered within the East Bay area of San Francisco, California, USA (Figure 1). These 2 isolates were from recurrent invasive GAS infections within the same patient that occurred 1.5 months apart.

Comparison of the *S. pyogenes gacA-L* cluster with the corresponding *gcc* loci from group C SE strains (SE-7136; Figure 2) revealed that GCS/SE genes shared homology with all 12 *gacA-L* genes (56%–89% sequence identity). The weakest conservation was observed between the *gac/gccIJK* genes (56%–69% identity), consistent with the requirement of *gacIJK* for the group A immunodominant *N-*acetylglucosamine side chain but not for synthesis of the polyrhamnose core (*1*). Two additional genes, designated *gccM* (glycotransferase gene) and *gccN* (UDP-monosaccharide epimerase gene), were evident within the *gcc* gene cluster. In the ancestral recombination event, an 11,527-bp GAS (*S. pyogenes*) chromosomal segment composed of the *gacE* 3′ portion, along with the *gacF-L* genes and a 5′ portion of *ebsA*, replaced the corresponding 13,813 bp of a GCS/ST128/SE strain, resulting in the recombinant GAS/ST128/SE lineage (Figure 2). This fragment encompasses the intact 7-gene *gacF–gacL* segment; each gene shared 99.4%–99.7% sequence identity with counterparts in *S. pyogenes*. The evident functionality of the hybrid *gac/gcc* cluster within the GAS/ST128/SE progeny lineage is consistent with identical roles of the first 3 genes of the cluster (*gac/gccA–C*) in the biosynthesis of the polyrhamnose core (*1*) that is present within the groups A, C, and G carbohydrates (*9*). Each of these 3 genes are also required for *S. pyogenes* viability (*1*).

The occurrence of multiple *emm* types within the same MLST is common in SE (*5*,*6*) and differs from *emm*/MLST associations within *S. pyogenes*, where an MLST is nearly always definitive of a single *emm* type (*2*,*10*). The presence of 3 different *emm* types and 8 macrolide-resistant isolates within GAS/ST128/SE (Figure 1) is indicative of a long-standing successful lineage. A single isolate of this lineage was positive for the exotoxin gene *speC* (Figure 1) that was carried on a prophage highly similar to a previously described *speC*-positive *S. pyogenes* strain (*8*). The relative genomic positions of the prophages are exactly conserved between the 2 species, inserted within the *pepD* gene in the genomic region that lies between the bacterial cell division genes *ftsE/ftsX* and the ribosomal protein gene *rpsL31B* (Figure 1). The number of single-nucleotide polymorphism differences between individual GAS/ST128/SE core genomes ranged from 0 to 613 (Figure 1). The GAS/ST128/SE strain AC-2713 recovered 20 years ago (*3*) is also indicative of a long-established lineage.

The 34 GAS/ST128/SE isolates for which information was available (32 from blood, 1 from a joint, and 1 from a surgical wound) recovered in ABCs since January 1, 2015, were recovered from older adults (age range 22–93 years; mean age 63 years) from 8 ABCs sites; most (85%) patients were men. Most patients had underlying medical conditions (data not shown), including 16 with diabetes, 15 with cellulitis (including 1 who had necrotizing fasciitis), 8 with pneumonia, and 6 with septic shock. One patient with bacteremia died.

## Conclusions

ABCs identifies invasive infections caused by GAS without identification of isolates to the species level. Since 2015, when we implemented WGS as our primary platform for GAS characterization, we have identified rarely occurring non–*S. pyogenes* isolates through our bioinformatics pipeline automated MLST function rather than previously employed phenotypic testing. Of ≈16,000 GAS isolates recovered from ABCs during 1994–2014, only 11 had *emm* types characteristic of SE. All 11 were collected during 2011–2014 and were of the 3 *emm* types found among the 35 GAS/ST128/SE isolates from this study. Genomic analysis verified the GAS/ST128/SE lineage of these 11 older isolates (data not shown). Finding 35 additional invasive isolates of this lineage recovered during January 1, 2015–November 1, 2018, through ABCs suggests a level of expansion attributable to strain adaptation and fitness or to a more susceptible population. Attempts to identify circulating ST128/SE strains of the original group C have been unsuccessful, including an examination of a population-based sampling of SE (*5*).

Because group A SE is suspected to be rare, these findings raise the question of whether invasive disease attributable to SE of groups C, G, and L is also increasing. A 2-year population-based study of β-hemolytic streptococcal disease attributable to Lancefield groups other than A and B within 2 ABCs sites during 2002–2004 revealed that 80% of such isolates were SE (*11*), with clinical manifestations and targeted susceptible populations similar to *S. pyogenes*. Incidence of invasive disease attributable to non–group A SE during this period was estimated at 2.5 cases/100,000 population, similar to the incidence of GAS infections (2.89 cases/100,000 population) in these same 2 sites. The incidence of overall invasive GAS disease in the United States has also markedly increased during recent years, from 3.4 cases/100,000 population in 2012 to 7.2 cases/100,000 population in 2017 (https://www.cdc.gov/abcs/reports-findings/survreports/gas17.html).

Appendix Additional information about emergent invasive group A *Streptococcus dysgalactiae* subsp. *equisimilis*, United States, 2015–2018.

## References

[1] van Sorge NM, Cole JN, Kuipers K, Henningham A, Aziz RK, Kasirer-Friede A, et al. The classical lancefield antigen of group a *Streptococcus* is a virulence determinant with implications for vaccine design. Cell Host Microbe. 2014;15:729–40. 10.1016/j.chom.2014.05.009 24922575 PMC4078075

[2] Chochua S, Metcalf BJ, Li Z, Rivers J, Mathis S, Jackson D, et al. Population and whole genome sequence based characterization of invasive group A streptococci recovered in the United States during 2015. MBio. 2017;8:e01422–17. 10.1128/mBio.01422-17 28928212 PMC5605940

[3] Brandt CM, Haase G, Schnitzler N, Zbinden R, Lütticken R. Characterization of blood culture isolates of *Streptococcus dysgalactiae* subsp. *equisimilis* possessing Lancefield’s group A antigen. J Clin Microbiol. 1999;37:4194–7. 10565964 10.1128/jcm.37.12.4194-4197.1999 PMC85928

[4] Tanaka D, Isobe J, Watahiki M, Nagai Y, Katsukawa C, Kawahara R, et al.; Working Group for Group A Streptococci in Japan. Genetic features of clinical isolates of Streptococcus dysgalactiae subsp. equisimilis possessing Lancefield’s group A antigen. J Clin Microbiol. 2008;46:1526–9. 10.1128/JCM.02188-07 18305132 PMC2292899

[5] Ahmad Y, Gertz RE Jr, Li Z, Sakota V, Broyles LN, Van Beneden C, et al. Genetic relationships deduced from *emm* and multilocus sequence typing of invasive *Streptococcus dysgalactiae* subsp. *equisimilis* and *S. canis* recovered from isolates collected in the United States. J Clin Microbiol. 2009;47:2046–54. 10.1128/JCM.00246-09 19386831 PMC2708495

[6] McMillan DJ, Bessen DE, Pinho M, Ford C, Hall GS, Melo-Cristino J, et al. Population genetics of *Streptococcus dysgalactiae* subspecies *equisimilis* reveals widely dispersed clones and extensive recombination. PLoS One. 2010;5:e11741. 10.1371/journal.pone.0011741 20668530 PMC2909212

[7] Lancefield RC. The antigenic complex of *Streptococcus haemolyticus*: I. Demonstration of a type-specific substance in extracts of *Streptococcus haemolyticus.* J Exp Med. 1928;47:91–103. 10.1084/jem.47.1.91 19869404 PMC2131344

[8] Walker MJ, Brouwer S, Forde BM, Worthing KA, McIntyre L, Sundac L, et al. Detection of epidemic scarlet fever group A Streptococcus in Australia. Clin Infect Dis. 2019; Epub ahead of print. 10.1093/cid/ciz099 30721938

[9] Coligan JE, Kindt TJ, Krause RM. Structure of the streptococcal groups A, A-variant and C carbohydrates. Immunochemistry. 1978;15:755–60. 10.1016/0161-5890(78)90105-0 85600

[10] Enright MC, Spratt BG, Kalia A, Cross JH, Bessen DE. Multilocus sequence typing of *Streptococcus pyogenes* and the relationships between *emm* type and clone. Infect Immun. 2001;69:2416–27. 10.1128/IAI.69.4.2416-2427.2001 11254602 PMC98174

[11] Broyles LN, Van Beneden C, Beall B, Facklam R, Shewmaker PL, Malpiedi P, et al. Population-based study of invasive disease due to beta-hemolytic streptococci of groups other than A and B. Clin Infect Dis. 2009;48:706–12. 10.1086/597035 19187026

