# Supplementary material for: Emergent Invasive Group A Streptococcus dysgalactiae subsp. equisimilis, United States, 2015–2018
Source: Emerg Infect Dis. 2019 Aug;25(8):1543–7. doi: 10.3201/eid2508.181758 (PMC6649341; doi:10.3201/eid2508.181758)
Supplement: Appendix — Additional information about emergent invasive group A Streptococcus dysgalactiae subsp. equisimilis, United States, 2015–2018. [file 18-1758-Techapp-s1.pdf]

Article DOI: https://doi.org/10.3201/eid2508.181758

Emergent Invasive Group A *Streptococcus dysgalactiae* subsp. *equisimilis*, United States, 2015–2018

Appendix

Construction of Maximum-Likelihood Trees

We constructed maximum-likelihood trees (Figure 1, https://wwwnc.cdc.gov/EID/article/25/8/18-1758-F1.htm) by using kSNP3.0 (*1*) with a kmer size of 19 and used MEGA 7 for evolutionary analysis (*2*). We inferred the evolutionary history by using the maximum-likelihood method on the basis of the general time reversible model (*3*). The trees with the highest log likelihood (−124033.37 for left tree and −13853.47 for right tree) are shown. We obtained initial trees for the heuristic search automatically by applying neighbor-join and BioNJ algorithms to a matrix of pairwise distances estimated using the maximum composite likelihood approach and then selecting the topology with superior log likelihood value (*2*). Multilocus sequence type, *emm* type determination, and grouping traits were determined as described previously (*4*–*6*). By using the CDC *S. pyogenes* whole-genome sequencing bioinformatics pipeline (*4*), we found that all but 7 of the strains shown were positive for the *sfb1* fibronectin binding protein gene (data not shown). Three of the 7 *sfb1*-negative strains included AC2713 and the 2 identical EB1–5 isolates. All strains except for the 2 group L strains (left tree) were positive for the query for the *S. pyogenes nga* determinant encoding the extracellular NAD+ glycohydrolase virulence factor (data not shown). As shown, 13 strains were positive for *erm*– or *mef*– encoded macrolide-resistance determinants, including a cluster of 8 GAS/ST128 isolates of *emm* subtype *stG245.0.* A single group *ermT*-positive group L strain had the ParC S79Y substitution associated with fluoroquinolone-resistance. In addition, a single group G/ST277 strain was positive for the pilus determinant T58. Single isolates were positive for the CDC pipeline queries for exotoxin genes *smeZ* (group L in left tree) and *speC* (GAS/ST128/*stG652.0*). All strains were β-hemolytic because of expression of the previously

characterized homologue of the *S. pyogenes* streptolysin S determinant (data not shown) (*7*). A total of 19,701 positions (for 49 isolates of left tree) and 2,108 (36 isolates of right tree) are included in the final datasets. The distance reference is indicative of 394 single nucleotide polymorphisms based on 19,701 positions within the 48 genomes (left tree) or 42 positions based upon 2,108 positions within 36 genomes (right tree). Genome fastQ accession numbers are provided for the 48 isolates (Appendix Table) and are associated with laboratory identifiers as well as the information provided from CDC *S. pyogenes* bioinformatics pipeline as described in Chochua et al. (*4*) and Appendix (Construction of Maximum-Likelihood Trees).

Appendix Table. SRA accession numbers, laboratory identifiers, and other features of *Streptococcus dysgalactiae* subsp. *equisimilis* study isolates

Biosample accession no.	LABID*	Group†	MLST	County, year‡	*emm* subtype§	Features detected from *S. pyogenes* WGS bioinformatics pipeline¶
SAMN09848964	20173110	A	128	NM2, 2016	*stG652.0*	*sfb1*, *nga*
SAMN09848983	20173524	A	128	NM2, 2017	*stG652.0*	*sfb1*, *nga*
SAMN07153383	20154682	A	128	NM2, 2015	*stG652.0*	*sfb1*, *nga*
SAMN08690859	20164183	A	128	NM1, 2016	*stG652.0*	*sfb1*, *nga*
SAMN08691925	20171692	A	128	NM3, 2016	*stG652.0*	*sfb1*, *nga*
SAMN09849571	20182387	A	128	NM3, 2017	*stG652.0*	*sfb1*, *nga*
SAMN09849325	20181492	A	128	NM4, 2017	*stG652.0*	*sfb1*, *nga*
SAMN07153783	20156439	A	128	EB1, 2015	*stG652.0*	*sfb1*, *nga*
SAMN07154238	20162102	A	128	EB2, 2015	*stG652.0*	*sfb1*, *nga*
SAMN08691109	20165290	A	128	EB1, 2016	*stG652.0*	*sfb1*, *nga*
SAMN08691691	20170764	A	128	NM3, 2016	*stG652.0*	*sfb1*, *nga*
SAMN08691143	20165326	A	128	CA1, 2016	*stG652.0*	*sfb1*, *nga*
SAMN08692346	20173686	A	128	EB2, 2016	*stG652.0*	*sfb1*, *nga*
SAMN08691545	20170128	A	128	CO1, 2016	*stG652.0*	*sfb1*, *nga*
SAMN07154260	20162127	A	128	CA1, 2015	*stG652.0*	*sfb1*, *nga*
SAMN08691583	20170168	A	128	CO2, 2016	*stG652.0*	*sfb1*, *nga*
SAMN08691937	20171717	A	128	OR2, 2016	*stG652.0*	*sfb1*, *nga*
SAMN09849664	20182677	A	128	OR1, 2017	*stG652.0*	*sfb1*, *nga*
SAMN07153796	20156452	A	128	EB1, 2015	*stG652.0*	*sfb1*, *nga*
SAMN07154052	20160976	A	128	NY2, 2015	*stG485.0*	*sfb1*, *nga*
SAMN08690593	20162522	A	128	GA5, 2016	*stG245.0*	*sfb1*, *nga*
SAMN08691696	20170850	A	128	TN1, 2016	*stG245.0*	*sfb1*, *nga*
SAMN07154389	20163069	A	128	CT2, 2015	*stG652.0*	*sfb1*, *nga*
SAMN08691781	20171173	A	128	GA2, 2016	*stG652.0*	*sfb1*, *nga, speC*
SAMN09849027	20176966	A	128	CO3, 2017	*stG485.0*	*sfb1*, *nga*
SAMN08691278	20165952	A	128	TN2, 2016	*stG245.0*	*sfb1*, *nga, ermB*
SAMN07153678	20156157	A	128	NY1, 2015	*stG245.0*	*sfb1*, *nga, mef*
SAMN07153302	20154376	A	128	NY3, 2015	*stG245.0*	*sfb1*, *nga, mef*
SAMN07153560	20155412	A	128	NY1, 2015	*stG245.0*	*sfb1*, *nga, mef*
SAMN09848910	20163223	A	128	NY1, 2016	*stG245.0*	*sfb1*, *nga, mef*
SAMN08691739	20170914	A	128	NY1, 2016	*stG245.0*	*sfb1*, *nga, mef*
SAMN08691478	20166757	A	128	NY2, 2016	*stG245.0*	*sfb1*, *nga, mef*
SAMN10342858	20185475	A	128	NY2, 2018	*stG245.0*	*sfb1*, *nga, mef*
SAMN07154253	20162120	A	128	EB1, 2015	*stG485.0*	*nga*
SAMN07154273	20162140	A	128	EB1, 2015	*stG485.0*	*nga*
SAMN09848906	20161329	G	48	NM3, 2015	*stG643.0*	*nga, mef*
SAMN09849191	20180052	G	127	MD2, 2017	*stG245.0*	*nga*, *tetM*
SAMN10342859	20162531	G	35	GA5, 2016	*emm23.4*	*sfb1*, *nga*, *mef*, *tetM*, *cat*
SAMN10342860	20170556	G	277	OR2, 2016	*stG6.0*	*nga*, *tee58*
SAMN08692801	20175240	C	20	NM3, 2017	*stG62647.0*	*nga*
SAMN08691915	20171682	C	17	NM1, 2016	*stC74A*.0	*sfb1*, *nga*, *ermTR*
SAMN09848934	20170560	G	17	OR3, 2016	*stG485.0*	*sfb1*, *nga*
SAMN08691738	20170893	G	206	CT1, 2016	*stG245.0*	*sfb1*, *nga*
SAMN08690595	20162524	G	17	GA1, 2016	*stG245.1*	*sfb1*, *nga*
SAMN09849033	20177043	G	17	GA3, 2017	*stG6792.0*	*sfb1*, *nga*
SAMN10342861	20185314	G	470	OR2, 2018	*stG840.0*	*sfb1*, *nga*
SAMN10342862	20161734	L	1	MD1, 2016	*stL1921.0*	*sfb1*, *smeZ*
SAMN10342863	20163969	L	467	MN1, 2016	*stL1929.1*	*sfb1*, *ermT*, ParC-S79Y

*These isolates are listed in same order (top to bottom) as in Figure 1, panel A (https://wwwnc.cdc.gov/EID/article/25/8/18-1758-F1.htm).
†The group A strains were positive for the group A carbohydrate cluster gene I (*gacI*). The group carbohydrate for these and the strains of groups C, G, and L were also determined serologically.
‡County and year of isolation. For example, NM2, 2016 refers to New Mexico county 2 and isolation during 2016.
§*emm* subtypes provided from CDC M protein gene database at ftp://ftp.cdc.gov/pub/infectious_diseases/biotech/tsemm.
¶Additional features obtained from CDC whole genome sequencing bioinformatics pipeline as described in Chochua et al. (*4*) and this Appendix (Construction of Maximum-Likelihood Trees).

References

1. Gardner SN, Slezak T, Hall BG. kSNP3.0: SNP detection and phylogenetic analysis of genomes without genome alignment or reference genome. Bioinformatics. 2015;31:2877–8. PubMed http://dx.doi.org/10.1093/bioinformatics/btv271

2. Kumar S, Stecher G, Tamura K. MEGA7: Molecular Evolutionary Genetics Analysis version 7.0 for bigger datasets. Mol Biol Evol. 2016;33:1870–4. PubMed http://dx.doi.org/10.1093/molbev/msw054

3. Nei M, Kumar S. Molecular evolution and phylogenetics. New York: Oxford University Press; 2000. p. 150–75.

4. Chochua S, Metcalf BJ, Li Z, Rivers J, Mathis S, Jackson D, et al. Population and whole genome sequence based characterization of invasive group A streptococci recovered in the United States during 2015. MBio. 2017;8:e01422–17. PubMed http://dx.doi.org/10.1128/mBio.01422-17

5. Lancefield RC. The antigenic complex of *Streptococcus haemolyticus*: I. Demonstration of a type-specific substance in extracts of *Streptococcus haemolyticus*. J Exp Med. 1928;47:91–103. PubMed http://dx.doi.org/10.1084/jem.47.1.91

6. Broyles LN, Van Beneden C, Beall B, Facklam R, Shewmaker PL, Malpiedi P, et al. Population-based study of invasive disease due to beta-hemolytic streptococci of groups other than A and B. Clin Infect Dis. 2009;48:706–12. PubMed http://dx.doi.org/10.1086/597035

7. Humar D, Datta V, Bast DJ, Beall B, De Azavedo JC, Nizet V. Streptolysin S and necrotising infections produced by group G streptococcus. Lancet. 2002;359:124–9. PubMed http://dx.doi.org/10.1016/S0140-6736(02)07371-3
